# Tropical bat ectoparasitism in continuous versus fragmented forests: A gap analysis and preliminary meta‐analysis

**DOI:** 10.1002/ece3.9784

**Published:** 2023-02-01

**Authors:** Alexis M. Heckley, Daniel J. Becker

**Affiliations:** ^1^ Department of Biology and the Redpath Museum McGill University Montreal Quebec Canada; ^2^ Department of Biology University of Oklahoma Norman Oklahoma USA

**Keywords:** Chiroptera, deforestation, ectoparasitism, land conversion, sampling bias, vector‐borne disease

## Abstract

Tropical regions are experiencing rapid rates of forest fragmentation, which can have several effects on wildlife, including altered parasite dynamics. Bats are a useful host group to consider the effects of fragmentation, because they are abundant in the tropics, serve important ecological roles, and harbor many parasites. Nevertheless, research on the effects of fragmentation on bat ectoparasites is still limited. To help guide ongoing and future research efforts, this study had two objectives: (1) conduct a gap analysis to characterize the state of currently available research on fragmentation effects on bat ectoparasites and (2) conduct a preliminary meta‐analysis to identify current trends. We systematically highlighted several research gaps: Studies comparing the effects of fragmented versus continuous forests on ectoparasites are limited and have primarily been conducted in the Neotropics, with a focus on bats in the superfamily Noctilionidea (especially frugivorous phyllostomids). Our preliminary meta‐analysis suggested that ectoparasite prevalence (but not the mean or variance in intensity) was higher in fragments than in continuous forests. Moreover, prevalence increased with increasing roost duration, and mean intensity was higher for bats with higher wing aspect ratios. Intensity variance was affected by an interaction between forest type and wing aspect ratio, such that variance increased for bats with high‐wing aspect ratios in continuous forests but decreased in fragments. These results suggest that fragmentation can shape aspects of bat ectoparasitism and could have implications for the ecology, health, and conservation of bats in fragmented landscapes. However, existing research gaps could bias our current understanding of habitat change and bat health, and future research should thus investigate these effects in the Paleotropics and with other bat families.

## Graphical abstract

We conducted a gap analysis and preliminary meta‐analysis to characterize the state of existing research on the relationship between forest fragmentation and bat ectoparasitism. We identified blatant knowledge gaps: Notably, current research is limited and has primarily been conducted with Neotropical bats, especially bats from the superfamily Noctilionidea. Our preliminary meta‐analysis suggests that ectoparasite prevalence (but not the mean or variance in intensity) is higher in fragments than in continuous forests and that this trend could be moderated by different bat traits—although more empirical work is needed to make concrete conclusions.

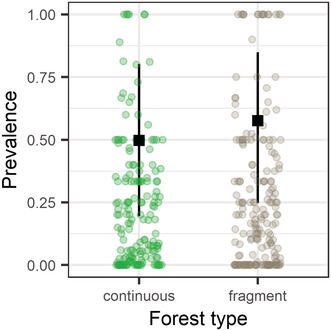

## 1 INTRODUCTION

Forest fragmentation can have severe effects on wildlife, ranging from reduced animal health (Janin et al., 2011) to altered population densities (Suzan et al., 2012) and increased extinction risks (Crooks et al., 2017). Wildlife is likely to be disproportionately impacted by these effects in the tropics, where forests are experiencing accelerated rates of fragmentation (Malhi et al., 2014). Indeed, from the years 2000 to 2012, tropical regions experienced the greatest loss of total forest globally (Hansen et al., 2013). The effects of fragmentation are thus especially pertinent to consider in these regions, and tropical wildlife risk facing substantial impacts.

Through these effects on wildlife, forest fragmentation can have important implications for parasites (Suzan et al., 2012). For example, individuals in fragmented habitats can have increased concentrations of baseline glucocorticoids (Messina et al., 2018), which can decrease immune function and increase susceptibility to infections (e.g., Woo et al., 1987). As another example, individuals will often congregate in remaining patches after a forest is fragmented (Suzan et al., 2012). Population densities can therefore be higher in fragmented forests than in continuous forests (Suzan et al., 2012), which can lead to a higher prevalence of density‐dependent parasites (McCallum, 2001). Finally, biodiversity loss is one of the most notable consequences of forest fragmentation (Haddad et al., 2015); and although biodiversity loss can in some cases reduce parasite prevalence, it is generally linked to higher infection prevalence and parasite transmission (Halliday et al., 2020; Keesing et al., 2010).

Bats are useful to study in the context of forest fragmentation and parasite infections for several reasons. First, bats are highly abundant and diverse, occupying almost every trophic level and roosting in a variety of structures and habitat types (Kunz & Fenton, 2005); as such, bats can be important indicators of habitat disturbance. Indeed, several studies have documented the responses of different bat species to fragmentation. These responses can range from individual‐ to ecosystem‐level impacts and have been found to vary by species, foraging ensemble, and the spatial scale of the study (Meyer et al., 2016). Second, as bats serve ecologically important roles, such as seed dispersal, pollination, and pest control, the impacts of forest fragmentation on bats can have cascading effects on other components of the environment (Kunz et al., 2011). Finally, bats harbor many parasites (including both micro‐ and macroparasites), several of which have received attention for their potential consequences to human health (Wang & Anderson, 2019), with notable examples including lyssaviruses (Schneider et al., 2009) and coronaviruses (Banerjee et al., 2019). Evidence even suggests some bat ectoparasites may act as vectors of zoonotic pathogens (e.g., bat flies and *Bartonella* spp.; Morse et al., 2012).

In the present analysis, we focus on tropical bat ectoparasites. Although studies are increasingly investigating how fragmentation can affect bat ectoparasites (e.g., Bolívar‐Cimé et al., 2018), research in this field is still limited. As with any emerging research topic, there is tremendous value in characterizing the state of currently available data and identifying knowledge gaps at a relatively early stage to help guide ongoing and future research efforts. Therefore, our primary aim was to conduct a gap analysis of the association between habitat types (continuous versus fragmented forests) and the prevalence and intensity of tropical bat ectoparasites.

As a secondary aim of this study, we also complemented our gap analysis with a preliminary meta‐analysis of the current data, with the goal that suggestive trends could help to prioritize future research. This meta‐analysis allowed for the identification of general patterns across species and regions, providing broader insight into the relationship between habitat comparisons (continuous versus fragmented forests) and ectoparasitism in this important host taxon. We predicted that ectoparasite prevalence and intensities would be higher in fragmented than in continuous forests. We also considered wing aspect ratio, maximum colony size, roost duration, and diet in our analyses, as we expected each of these variables could impact the magnitude of the effects of fragmentation. For example, bat flies are common ectoparasites that pupate in roosting structures and then search for bat hosts after developing into their adult stage (Dick & Patterson, 2006; Patterson et al., 2007). Given that bat fly transmission is highly dependent on the roost, their prevalence and intensities have been associated with roost duration (Patterson et al., 2007). Through potential damages to roosting structures (e.g., trees), forest fragmentation could therefore impact this association between roost duration, prevalence, and intensity for this common ectoparasite group. As another example, the wing aspect ratio has been associated with environmental filtering in fragmented versus continuous forests, presumably because bats with low wing aspect ratios are well adapted for maneuvering in dense forest and thus perform poorly in fragments, whereas bats with high wing aspect ratios retain high mobility (Farneda et al., 2015). Ectoparasite outcomes could thus differ in fragmented and continuous forests due to wing aspect ratios filtering bat host responses to fragmentation; if bats with low wing aspect ratios are not well adapted to fragments, those species would have fewer opportunities for ectoparasite transmission in fragmented habitats.

## 2 METHODS AND MATERIALS

### 2.1 Search protocol

To investigate the relationship between forest fragmentation and ectoparasite prevalence and intensity in tropical bats, we searched Web of Science, CabDirect, and PubMed on January 24, 2022, using the following terms: [(bat* OR chiroptera) AND (infection OR parasit* OR prevalence OR ectoparasit* OR intensit*) AND (deforest* OR degrad* OR fragment* OR land conver* OR land clear* OR land use)]. For Web of Science, we searched for Topic field tags (i.e., the “TS” field tag), and for CabDirect and PubMed we searched all fields. For only PubMed, we slightly modified the above search terms to start with “bat OR bats OR chiroptera” due to their system preventing the use of asterisks for words with fewer than four letters. These searches resulted in 2533 studies across databases (2068 records from Web of Science, 354 from PubMed, and 111 from CabDirect), or 2360 once we removed duplicates. Following a systematic protocol (Moher et al., 2009), we then filtered abstracts to only include studies that met our inclusion criteria: (1) be conducted in the tropics, (2) provide prevalence data for any ectoparasite infesting any bat species (or the data are presented in a way where this can be calculated), and (3) consider at least one disturbed site (i.e., fragmented or degraded) and one nondisturbed site (i.e., continuous forest). Fragments in urban areas or known tourist destinations (e.g., caves that are described as being frequented by tourists) were not included due to the potential for confounding effects. 2290 studies were excluded during the initial abstract screening phase, where we excluded studies that were not conducted in the tropics or with bats, or that did not look at parasites. The remaining 70 studies were looked at fully. All abstracts were screened, and data were extracted, by one person (AH). Nine studies met the inclusion criteria (a list of data sources used in the study are provided in Appendix S1).

Most studies that met our inclusion criteria provided clearly demarcated habitats as either fragmented or continuous forests, or were sampled along gradients where the extremes could be classified as either fragmented or continuous. However, the habitats in one study did not align as closely with these categories (Orta‐Pineda et al., 2020). Here, bats were sampled along a disturbance gradient, ranging from fully disturbed pasture to fallow land that included shrubs, trees, and grasses. We retained this study in our analyses because (1) fallow land could be considered continuous forest depending on the time since clearance, and (2) almost all studies retained in our analyses describe some degree of human disturbance, even in the continuous forests (Appendix S2, Table A1). For the gap analysis, the inclusion of Orta‐Pineda et al. (2020) highlights the importance of quantitative measures of habitat characteristics in assigning fragmentation categories. For the meta‐analysis, we included this study in the primary analyses below and provide results with it excluded in the Supporting Information (Appendix S2); however, the inclusion or inclusion of this study did not result in dramatically different trends (see Sensitivity to the influential study in Results).

Similarly, given that the ectoparasites in our dataset were either bat flies or mites (and were heavily dominated by the former; see Bat and parasite species in Results), our primary analyses pooled all ectoparasite taxa. However, because mites can differ in infestation intensity and other traits (e.g., host specificity) that might impact relationships between fragmentation and ectoparasitism (e.g., Reeves et al., 2016), we also provide results from primary analyses with mites excluded in the Supporting Information (Appendix S2).

### 2.2 Gap analysis

To identify the state of currently available data, we first highlighted where studies have been conducted on habitat fragmentation and bat ectoparasites by mapping the countries represented in our dataset. Next, we used phylogenetic comparative methods to assess sampling effort among bat species. We used the *ape* package v 5.4.1 (Paradis et al., 2004) to trim a recent mammal supertree to bats (Upham et al., 2019), representing the majority (*n* = 1287) of the over 1400 recognized species in this order (Simmons & Cirranello, 2022). We differentiated bat species that have been studied in our dataset from nonstudied bat species and used the *caper* package to estimate the *D* statistic as a measure of the phylogenetic signal in binary sampling effort (Fritz & Purvis, 2010; Orme et al., 2010). Significant departure from a Brownian motion model of evolution (*D* = 1) and a phylogenetically random model (*D* = 0) was quantified with a randomization test. Next, we applied a graph partitioning algorithm, phylogenetic factorization, to flexibly identify bat clades across taxonomic levels that differ in the fraction of species studied. Phylogenetic factorization partitions a phylogeny by iteratively identifying edges in a tree that maximizes an objective function contrasting species separated by the edge. We used the *phylofactor* package here to partition sampling effort as a Bernoulli‐distributed response in a generalized linear model (Washburne et al., 2019). We determined the number of phylogenetic factors (clades) to retain using Holm's sequentially rejective 5% cutoff for the family‐wise error rate (Holm, 1979).

### 2.3 Preliminary meta‐analysis

#### 2.3.1 Data extraction

For the meta‐analysis, we next had to extract data from the studies that met the inclusion criteria. In three cases, we extracted data from the text, supplementary material, or a digital repository. In another four cases, we e‐mailed the authors for data, resulting in the exclusion of two studies owing to no response. In two studies, the authors described sampling in different locations, including towns or sites affected by urbanization but did not provide site‐specific sampling information (i.e., we were unable to exclude bats collected in towns or cities). These studies were excluded. We, therefore, included five studies in our analyses (Appendices S1, S2, Figure A1), with each unique observation in our dataset representing, from each study, a given bat species, captured at a given site, infested with a given parasite (to the lowest taxonomic resolution provided by the authors or that we were able to discern from the available data). Although five is a relatively small number of studies for meta‐analyses in ecology (Koricheva et al., 2013), similar sample sizes are common in other fields, particularly in medical research (e.g., Bell et al., 2018, *n* = 4; Fransson et al., 2018, *n* = 3; Fridh et al., 2021, *n* = 4). Further, because most studies in our analyses sampled multiple bat species in multiple locations, our dataset consisted of a robust number of unique observations (prevalence: *n* = 411; intensity: *n* = 116). Nevertheless, owing to the small number of studies, any trends in our meta‐analysis are only suggestive and findings should be interpreted as preliminary. The limited number of studies reflects the lack of research in this field, and we hope that our gap‐ and meta‐analysis will inspire the additional empirical work needed for future meta‐analyses to identify more concrete patterns.

In addition to prevalence data, we also extracted intensity data if provided (i.e., mean parasite counts), the country of the study sites, the nature of the habitat fragmentation (e.g., for agriculture, anthropogenic island, etc.), and the bat and parasite species. To filter through studies, we used the *metagear* package v 0.7 in R (Lajeunesse, 2016; R Development Core Team, 2021) and the Rayyan web platform (Ouzzani et al., 2016).

#### 2.3.2 Bat trait data

We then compiled external data for species‐level traits that we hypothesized could impact the relationship between habitat fragmentation and parasite prevalence and/or intensity. These traits included bat taxonomy (Upham et al., 2019), diet (Wilman et al., 2014), roost duration (Patterson et al., 2007), wing aspect ratio (Norberg & Rayner, 1987), and maximum colony size (Santana et al., 2011). We complemented these data with additional literature searches when information was lacking for a specific bat species in our dataset. Given differences in trait presentation among datasets, we converted some continuous variables into categories. For diet, we grouped bat species as frugivores, sanguivores, nectarivores, carnivores, or insectivores, depending on whether >50% of their diet consisted of fruit, blood, nectar, animals, or insects, respectively (Becker, Chumchal, et al., 2018; Wilman et al., 2014); diet was categorized as a variable for species whose diets did not comprise more than 50% of one food type. For colony size, we categorized maximum colony size as small (0–100 individuals), or medium‐large (100+ individuals), which is consistent with colony size ranges presented in similar analyses (e.g., Becker et al., 2020).

In addition to collating ecological traits as moderator variables, we also included bat phylogeny to control for potential phylogenetic dependence in our prevalence and intensity data. We used the *ape* package to trim the above‐described mammal supertree to our included bat species (Upham et al., 2019), and we computed a correlation matrix to use the phylogenetic information in subsequent analyses.

#### 2.3.3 Calculating outcome variables

Because prevalence is a proportion, and as the data were highly right‐skewed, we used logit‐transformed proportions as the outcome variable for prevalence. For intensity, we first used log‐transformed raw means to assess *mean* intensity, and we then calculated the log‐transformed ratio of the standard deviations to assess the *variance* of intensity (Nakagawa et al., 2015). The outcome variables and sampling variances for both the prevalence and intensity data were calculated using the escalc() function in the *metafor* package v 3.0.2 (Viechtbauer, 2010). These, and all subsequent analyses, were conducted in R v 4.0.4 (R Development Core Team, 2021).

#### 2.3.4 Statistical analyses

To first quantify the amount of heterogeneity in our three parasite outcome variables (ectoparasite prevalence, mean intensity, intensity variance), we built intercept‐only hierarchical meta‐analysis models to estimate *I* ^2^. This statistic quantifies the contribution of true heterogeneity (rather than noise) to variance in response variables (Senior et al., 2016). Models were fit using the rma.mv() function with the Quasi‐Newton BFGS optimizer in the *metafor* package and included weighting by sampling variance. We included an observation‐level random effect nested within a study‐level random effect to account for within‐ and between‐study heterogeneity (Konstantopoulos, 2011). A random effect for species was also included to account for repeat observations per bat species alongside a separate random effect for bat phylogeny that used our phylogenetic correlation matrix (Cinar et al., 2020). These two random effects account for two separate things in the analysis; the inclusion of species accounts for potential pseudoreplication in the event that some species are sampled more than once, whereas phylogeny accounts for the extent to which relatedness impacts the relationship between forest fragmentation and the outcome variables (Nakagawa & Santos, 2012). In addition to estimating heterogeneity in each response variable, we derived *I* ^2^ for models with forest type as a moderator to characterize heterogeneity in the relationship between forest fragmentation and parasite outcome variables. For all models, we calculated the total *I* ^2^ and a unique *I* ^2^ for each random effect. We considered heterogeneity to be low, moderate, or high corresponding to *I* ^2^ values of 25%, 50%, or 75%, respectively (Higgins, 2003). We also calculated the phylogenetic signal, *H* ^2^, for each model to assess phylogenetic relatedness among outcome variables and effect sizes. A low *H* ^2^ value suggests little relatedness among response variables for closely related species (Nakagawa & Santos, 2012). We fit models using restricted maximum likelihood (REML).

We next constructed three sets of hierarchical meta‐analysis models to separately investigate associations between habitat fragmentation and each parasite outcome variable. First, we constructed a full model with all possible moderator variables. For the prevalence model, no moderators displayed strong collinearity, and so, all moderators were included. For the intensity models, different roost duration values were biased towards different maximum colony size categories (e.g., bats with small maximum colony sizes tend to roost in less permanent roosting structures whereas bats with large colony sizes tend to roost in more permanent structures). Similarly, different traits were biased towards different diet categories (e.g., frugivores tend to use less permanent roosting structures, whereas sanguivores only use more permanent structures). As such, for intensity models, we retained roost duration but not maximum colony size (owing to fewer missing values in the dataset for roost duration), and the primary diet categories were collapsed into simply “frugivore” and “non‐frugivore.” As we were primarily interested in the relationship between forest type (i.e., fragmented or continuous) and parasite prevalence or intensity, all moderators were considered in interactions with forest type. We then simplified models by considering all possible combinations of the moderator variables and selecting the best of these models using Akaike information criterion (AIC; i.e., the model with the lowest AIC out of all possible models). This model selection procedure follows best practices by comparing among an a priori set of candidate models (Burnham & Anderson, 2004). Prevalence models were compared using AIC and, due to the smaller sample sizes, intensity models were compared using corrected AIC (AICc). Models were considered competitive within two ΔAIC(c) of the best‐fit model (Burnham & Anderson, 2004). Our candidate models were first fit to a dataset free of all missing values to facilitate ML comparison. For the most parsimonious model, we then refit the model with REML to the full dataset to best estimate model parameters. Intensity models had no missing values for either model fitting or the final models, but candidate models were still fitted with ML and final models with REML.

### 2.4 Sensitivity analysis

When we conducted a Cook's distance test clustered by study (using the *metafor* package), Orta‐Pineda et al. (2020) was identified as influential; however, this study (and one other influential study; Frank et al. (2016)), each contributed more than five times more data records than the third largest study in our dataset. We thus conducted a secondary series of analyses identical to our primary analyses with Orta‐Pineda et al. (2020) excluded. Complete results for this second analysis are provided in Appendix S2 (Table A3–A7). Similarly, we conducted an analysis with only bat flies included (i.e., with mites excluded), and these results are also provided in Appendix S2 (Tables A8–A12). For both of these analyses, notable results are presented in‐text (see: Sensitivity to the influential study and to the ectoparasite group in Results).

## 3 RESULTS

### 3.1 Gap analysis

#### 3.1.1 Study locations

**FIGURE 1:**
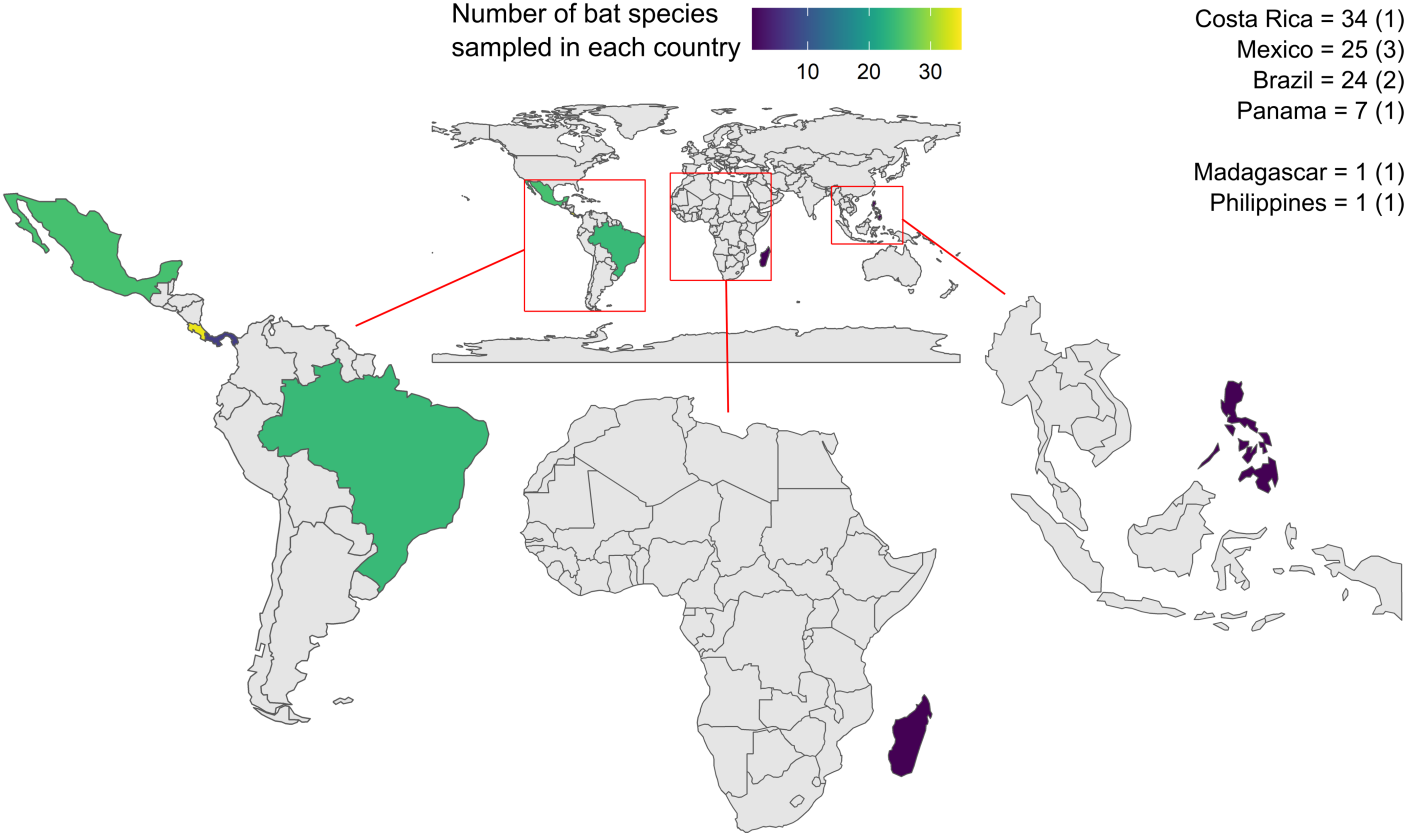
Countries where bats in the gap analysis studies were sampled. The color illustrates the number of unique bat species sampled in each country (the exact number of bat species sampled per country is provided on the top right, with the value in parentheses indicating the number of studies that sampled bats in that country).

Seventy‐eight percent of studies (*n* = 7) that compared bat ectoparasite measures between continuous and fragmented forests were conducted in the Neotropics. Neotropical bats were sampled in four countries: Mexico, Panama, Costa Rica, and Brazil (Figure 1). Paleotropical studies were conducted in two countries: the Philippines and Madagascar. Although all studies included both continuous and fragmented sites, fragments differed in whether they were used by humans or not. In three studies, the fragmented sites were used by humans (specifically, for agriculture or coffee plantations), and in four studies, researchers sampled sites used by humans and those not used by humans. Two studies did not specifically describe the habitats where bats were captured but described a range of human and nonhuman disturbances in the area.

#### 3.1.2 Bat and parasite species

Sixty‐two bat species were sampled in the context of habitat fragmentation and ectoparasitism, representing an extremely small fraction of bat diversity (<5%). In the Neotropics (*n* = 60; 97% of all sampled bat species), most of these species were from the family Phyllostomidae (49 species; 82%). The other four bat families were the Mormoopidae (3 species; 5%), Vespertilionidae (6 species; 10%), Molossidae (1 species; 2%), and Thyropteridae (1 species; 2%). In the Paleotropics (*n* = 2; 3% of all sampled bat species), the species included *Hipposideros diadema* from the Hipposideridae and *Myzopoda aurita* from the Myzopodidae. Forty‐four percent of bat species were frugivores (*n* = 27), 31% were insectivores (*n* = 19), 3% were nectarivores (*n* = 2), and 3% were sanguivores (*n* = 2). The remaining 19% had variable diets (*n* = 12).

**FIGURE 2:**
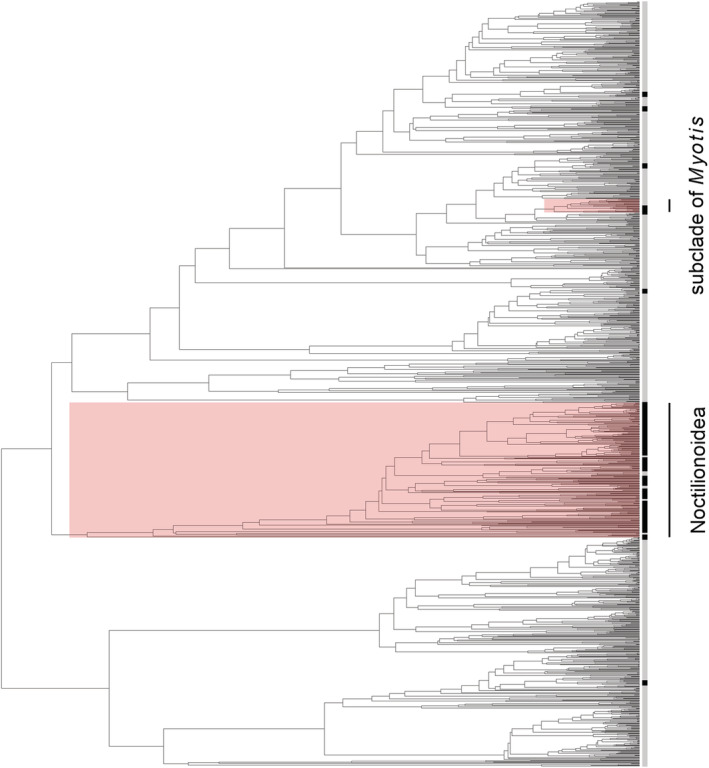
Phylogeny of bat species (Upham et al., 2019). Red shading highlights clades of bats identified as overrepresented in our dataset via phylogenetic factorization.

We detected an intermediate phylogenetic signal for whether bat species have been sampled for ectoparasites in the context of habitat comparisons (*D* = 0.68) (Figure 2). This phylogenetic pattern departed from both randomness (*p* < 0.001) and Brownian motion (*p* < 0.001). Phylogenetic factorization identified two clades with different sampling efforts, both of which were more heavily sampled than the rest of the bat phylogeny. Unsurprisingly given the above geographic patterns, the superfamily Noctilionidea has been more heavily sampled (24% of species, compared with 0.07% for the rest of the tree). However, the New World subclade of the *Myotis* genus has also been more heavily sampled (13%).

At least 63 ectoparasite species were sampled, although several studies did not report the specific parasite species, and in one study no ectoparasites were found. Sixty‐two known species belonged to the Hippoboscoidea superfamily (bat flies), as well as the families Spintunicidae (mites) and Spelaeorhynchidae (mites). Some authors also reported ectoparasites of the order Acari (representing at least one other species), but these were not identified to a finer taxonomic resolution.

### 3.2 Preliminary meta‐analysis

#### 3.2.1 Dataset description

Our meta‐analysis dataset consisted of 411 observations (representing a given bat species, from a given site, infested with a given parasite) from five studies that assessed the effects of forest fragmentation on ectoparasite prevalence in Neotropical bats. Studies were conducted in all four Neotropical countries represented in our gap analysis (Mexico, Panama, Costa Rica, and Brazil). Slightly under half of our dataset represented prevalence records from continuous forests (47%, *n* = 193), and 53% of our data were from fragments (*n* = 218). Most records from the fragmented areas were collected from land that serves a purpose for humans (*n* = 180; 83%). The fragments that were not used by humans (*n* = 38; 17%) were either anthropogenic islands or areas described as fragmented or degraded by the original authors; these fragments also had low forest cover or were small in size relative to other forested areas. Additionally, three studies provided intensity data. Our dataset therefore also consisted of 116 observations for ectoparasite intensity, from studies conducted in Costa Rica, Mexico, and Panama. From these, most sites were located in fragments (*n* = 64; 55%) that serve a purpose for humans (*n* = 52; 81%).

Most of the 49 different bat species in our prevalence dataset were from the family Phyllostomidae, representing 96% of observations (*n* = 394). The other two bat families were the Mormoopidae (2% of observations; *n* = 9) and Vespertilionidae (2% of observations; *n* = 8). Of these observations, 75% represented frugivorous bats (*n* = 307), 15% were from bats with variable diets (*n* = 60), 6% were from insectivores (*n* = 25), 4% were from sanguivores (*n* = 15), and 1% from were nectarivores (*n* = 4). The intensity dataset consisted almost exclusively of phyllostomid bats (98%, *n* = 114), but one vespertilionid was also represented (*Myotis keaysi;* 2%, *n* = 2), and most observations were similarly from frugivorous bats (79%, *n* = 92). Non‐frugivorous records included sanguivores (4%, *n* = 5), insectivores (3%, *n* = 3), and bats with variable diets (14%, *n* = 16). When considering ectoparasites, among the observations where the broader parasite group was known (i.e., bat flies or mites), almost all ectoparasites in our dataset were bat flies (90% in the prevalence dataset, *n* = 352; 88% in the intensity dataset, *n* = 97).

#### 3.2.2 Heterogeneity in parasite outcomes

When we considered intercept‐only models to obtain estimates of heterogeneity in our response variables, we found high *I* ^2^ for all three parasite outcomes (Table 1). Random effects of study and bat species had low heterogeneity in all models; the observation‐level random effect had low heterogeneity for mean intensity and low‐to‐moderate heterogeneity for intensity variance and prevalence. For phylogeny, we found moderate‐to‐high heterogeneity for prevalence and intensity variance; and high heterogeneity for mean intensity (Table 1). Finally, the phylogenetic signal for our intercept‐only models was low‐to‐moderate for ectoparasite prevalence and intensity variance but moderate‐to‐high for mean intensity (Table 1).

**TABLE 1:** Estimated heterogeneity (*I* ^2^) in parasite outcomes and phylogenetic signal (*H* ^2^) from each intercept‐only model. Values are presented as proportions.

*I* ^2^ term	Prevalence (*n* = 411)	Mean intensity (*n* = 116)	Intensity variance (*n* = 116)
Study	0.06	0.00	0.00
Observation	0.25	0.18	0.37
Bat species	0.00	0.00	0.00
Bat phylogeny	0.59	0.77	0.59
Total *I* ^2^	0.89	0.95	0.96
*H* ^2^	0.28	0.57	0.31

*Note*: The sample sizes of the data used in each model are indicated in parentheses next to the header.

**FIGURE 3:**
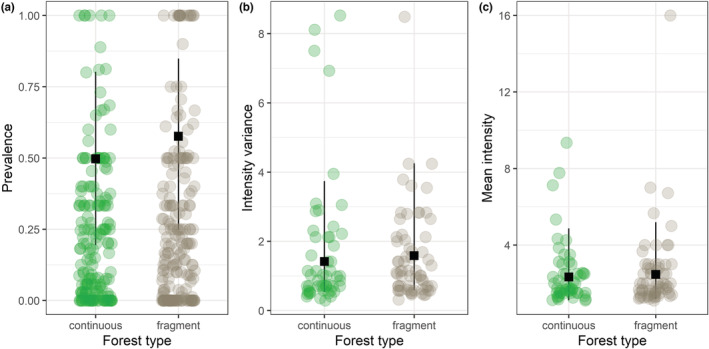
Forest type–only models for each parasite outcome, where prevalence is the proportion of infested individuals and intensity is the number of parasites per infested individual. The black squares represent values predicted from the final model with 95% confidence intervals, and the circles represent raw data (scaled by inverse sampling variance).

When forest type was considered as an independent moderator variable, we found a significant difference between fragmented and continuous forests for ectoparasite prevalence, where prevalence was higher in fragmented than in continuous forests (Table 2; Figure 3a). We did not observe an effect for either mean intensity or intensity variance (Table 2; Figure 3b,c). For these univariate forest‐type models, we again found high heterogeneity in effect size across all response variables (Table A3). Random effects of study and bat species again had consistently low heterogeneity; the observation‐level random effect had low heterogeneity for prevalence and mean intensity, and low‐to‐moderate heterogeneity for intensity variance; and phylogeny had moderate‐to‐high heterogeneity for prevalence and intensity variance, and high heterogeneity for mean intensity (Table A3). Phylogenetic signal was moderate‐to‐high for prevalence and intensity variance, and high for mean intensity.

**TABLE 2:** Summary of each forest‐type‐only model.

		*β*	SE	*z*	*p*	Lower 95% CI	Upper 95% CI
Prevalence	Intercept	−0.01	0.72	−0.02	0.99	−1.42	1.40
	**Fragment**	**0.32**	**0.13**	**2.53**	**0.01**	**0.07**	**0.57**
Intensity variance	Intercept	0.35	0.50	0.70	0.48	−0.62	1.32
	Fragment	0.11	0.14	0.80	0.42	−0.16	0.39
Mean intensity	**Intercept**	**0.85**	**0.38**	**2.25**	**0.02**	**0.11**	**1.58**
	Fragment	0.06	0.08	0.73	0.46	−0.09	0.21

*Note*: Bold font indicates a significant (<0.05) *p*‐value. Random effects for each model include observation nested within the study, bat species, and bat phylogeny.

#### 3.2.3 Moderators of relationships between forest type and parasite outcomes

To better understand the relationship between forest fragmentation and parasite outcomes, we next considered the effects of several moderator variables. Model comparison suggested that the interactions between forest type and wing aspect ratio, and forest type and roost duration (forest type: wing aspect ratio + forest type: roost duration), were the strongest predictors of ectoparasite prevalence (ΔAIC = 0, *w*_*i*_ = 0.80). When considering ectoparasite intensity, the interactions between forest type and wing aspect ratio, and forest type and diet (forest type: wing aspect ratio + forest type: diet) were the most parsimonious predictors of variance (ΔAIC = 0.002, *w*_*i*_ = 0.327). These same interactions were the best predictors of mean intensity (ΔAIC = 0, *w*_*i*_ = 0.34). For intensity variance, one other model was also competitive but had less Akaike weight (*w*_*i*_ = 0.26), and for mean intensity, two models were competitive but had less weight (*w*_*i*_ = 0.25, *w*_*i*_ = 0.16) (Appendix S2, Table A2).

**FIGURE 4:**
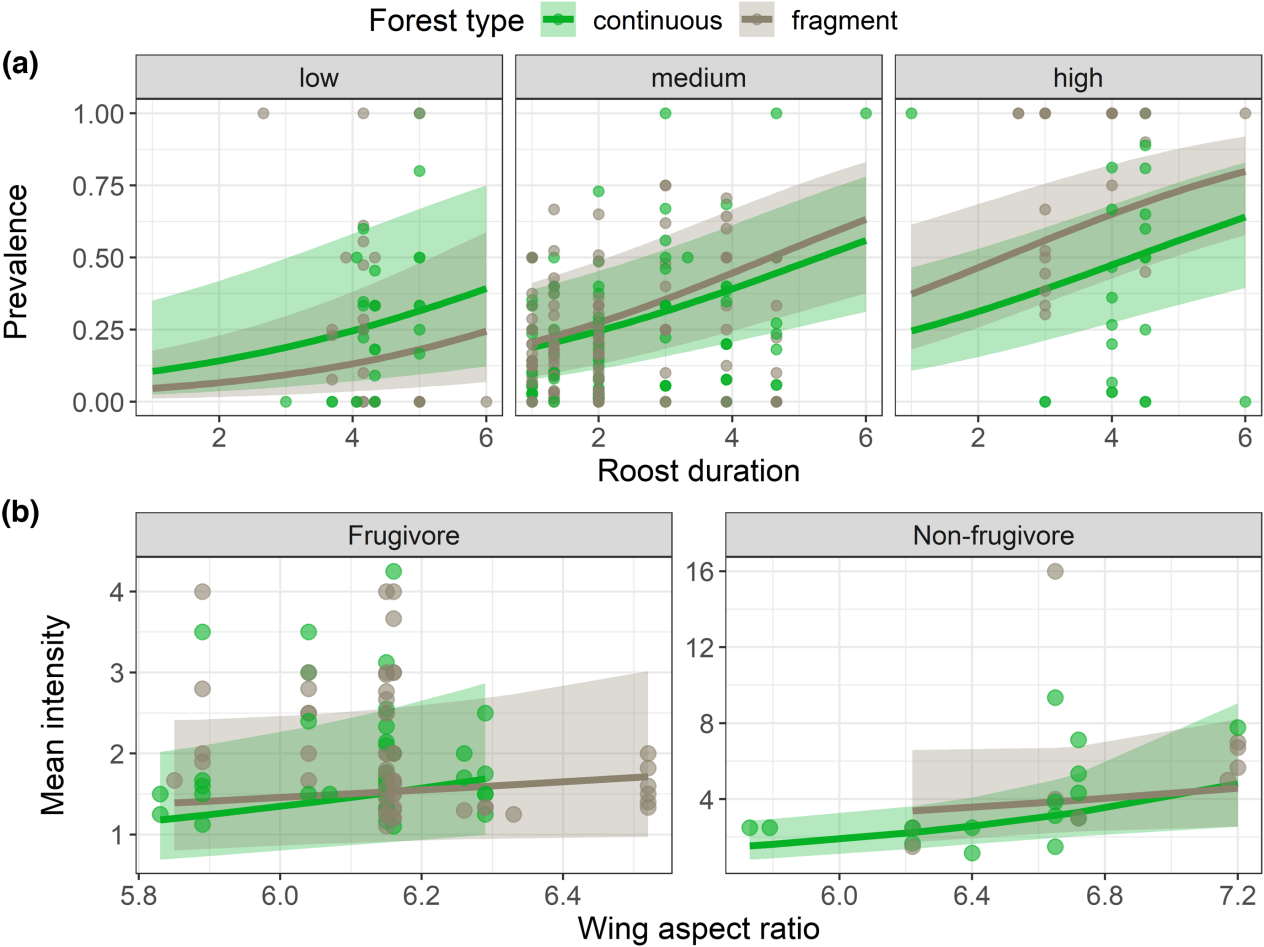
The effects of (a) roost duration and wing aspect ratio (low, medium, high) on prevalence (the proportion of infested individuals) in continuous and fragmented forests, and (b) the effect of wing aspect ratio on the mean intensity (the number of parasites per infested individual) of ectoparasite infestations in fragmented and continuous forests. The lines and ribbons represent values predicted from the final model with 95% confidence intervals, and the circles represent the raw data points. Prevalence values were predicted with no missing values (*n* = 292), and mean intensity values were predicted with missing values included (*n* = 103).

**FIGURE 5:**
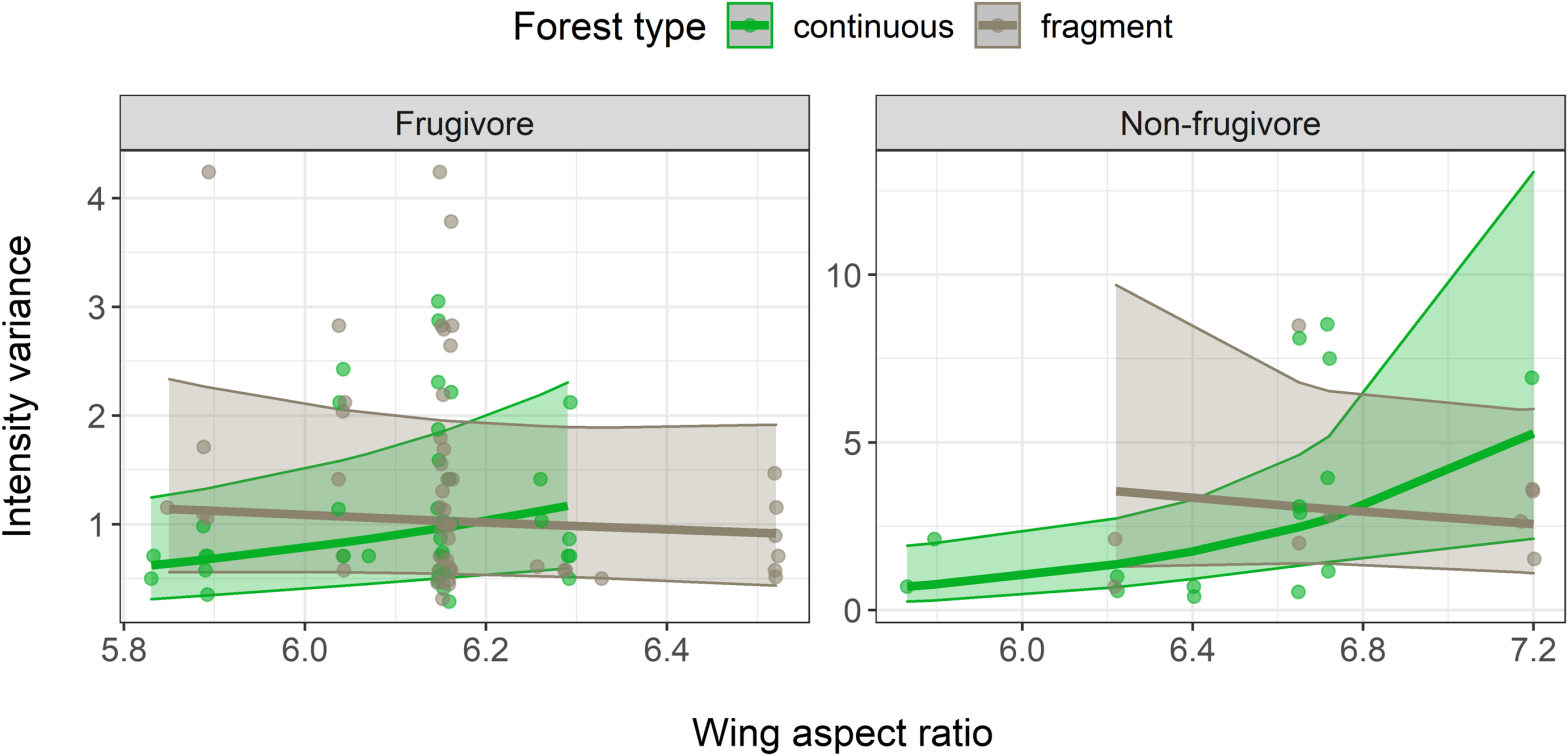
The effects of wing aspect ratio and primary diet in fragmented and continuous forests on the variance of ectoparasite intensity (the number of parasites per infested individual). The lines and ribbons represent values predicted from the final model with 95% confidence intervals, and the circles represent raw data. Intensity values were predicted with missing values included (*n* = 116). Note that the y‐axes are presented on different scales.

We found a significant effect of roost duration on ectoparasite prevalence, where prevalence increased with increasing roost duration (*β* = 0.34, CI = [0.14–0.53], *p* = 0.0006; Figure 4). For the intensity models, wing aspect ratio affected mean intensity (*β* = 0.78, CI = [0.19–1.37], *p* = 0.0100), and forest type and wing aspect ratio had a significant interactive effect on intensity variance (forest type: wing aspect ratio: *β* = 0.34, CI = [0.14–0.53], *p* = 0.0006). In short, irrespective of habitat fragmentation, bats with higher wing aspect ratios had a higher mean intensity (Figure 4), whereas bats with higher wing aspect ratios had higher intensity variance in continuous forests but lower intensity variance in fragments (Figure 5).

#### 3.2.4 Sensitivity to the influential study and to the ectoparasite group

When we excluded data from Orta‐Pineda et al. (2020; see Materials and Methods), we found a weaker overall effect of forest fragmentation on all parasite outcomes. Roost duration was still an important and significant predictor for prevalence. The interaction between forest type and wing aspect ratio was still an important predictor for intensity variance (an interaction between forest type and the diet category was also significant). Wing aspect ratio was also still important for mean intensity, although a model with only the interaction between forest type and primary diet was also highly competitive. Full results of this analysis are provided in the Supporting Information (Appendix S2, Tables A3–A7).

When excluding mites (*n* = 59), results were mostly consistent with the analyses above. Although the effect of fragmentation on all parasite outcomes was weaker (but the effect of forest type on prevalence was marginally significant; *p* = 0.05), roost duration remained an important predictor for prevalence; the interaction between forest type and wing aspect ratio remained important for intensity variance (roost duration was also significant, as well as an interaction between forest type and diet); and wing aspect ratio remained important for mean intensity (Appendix S2, Tables A8–A12).

## 4 DISCUSSION

Tropical regions globally are experiencing tremendous rates of forest fragmentation (Hansen et al., 2013; Malhi et al., 2014), and studies have increasingly investigated the effects of fragmentation on tropical wildlife (e.g., Crooks et al., 2017; Janin et al., 2011). In the present study, we conducted a gap analysis to highlight limitations in our understanding of the association between forest fragmentation and tropical bat ectoparasites. To identify interesting trends that might inspire future research, we also conducted a preliminary meta‐analysis of the existing literature. Below, we discuss data biases identified by our literature search and provide possible explanations for some of the trends observed in our meta‐analysis (although we note that these results must be interpreted with caution, owing to the limited sample size). We end by discussing the importance of providing quantitative measures of habitat characteristics, which will help facilitate the inclusion of more studies in future meta‐analyses.

### 4.1 Gap analysis

Our literature search revealed several biases in the current literature on tropical bats, habitat fragmentation, and ectoparasites. Most obviously, we only identified nine studies that directly compared bat ectoparasite outcomes between fragmented and continuous forests across the global tropics, highlighting a substantial lack of research in this area. Moreover, these explicitly comparative studies have primarily been conducted in the Neotropics (78% of studies, representing 97% of bat species in the gap analysis). This bias is consistent with past suggestions that the effects of forest fragmentation on bats are understudied in the Paleotropics, and we join other researchers in emphasizing a need for more research (including but not limited to effects of fragmentation) to be conducted in Paleotropical regions (Meyer et al., 2016).

Another notable bias concerns the bat species in our dataset. For instance, frugivores, the most heavily represented group (45% of unique species), are one of the most tolerant dietary guilds to habitat disturbance (Carballo‐Morales et al., 2021). If ectoparasite dynamics are less severely affected when infesting more tolerant species, this bias could result in conservative estimates of the effects of fragmentation. Moreover, the bat species in our dataset were primarily from the family Phyllostomidae (79%). Although this bias is common because most phyllostomid bats are more easily captured using typical sampling methods (e.g., ground‐level mist nets) than other bat families (Simmons et al., 1998), ecological or evolutionary differences between bat families could affect how the bats or their parasites respond to fragmentation. This bias is reflected in our phylogenetic analysis, where the superfamily Noctilionidea (which includes the family Phyllostomidae) has been more heavily sampled than other bat species. Interestingly, however, a subclade of New World *Myotis* has also been more heavily sampled than other bats, despite only representing a few species in our dataset (*n* = 4: *Myotis keaysi*, *M. nigricans*, *M. riparius*, *M. lavali*). Nevertheless, we suggest that future research comparing continuous and fragmented habitats focuses on a broader range of bat species (in particular, beyond frugivorous phyllostomid bats).

### 4.2 Meta‐analysis

To identify early trends that could inspire future research priorities, we conducted a preliminary meta‐analysis with five studies identified through our literature search. Overall, our results suggest that forest fragmentation is associated with greater ectoparasite prevalence and interacts with wing aspect ratio to impact intensity variance, but it may have little to no association with mean ectoparasite intensities. When we considered the effects of moderator variables relating to bat ecology, roost duration was an important predictor of prevalence, and wing aspect ratio was important for both intensity outcomes. These results suggest that forest fragmentation could affect some aspects of Neotropical bat parasitism and that some relationships may differ across distinct groups of bat species; however, more research is again needed to understand these effects.

When we only considered forest type in our meta‐analysis models, ectoparasite prevalence was higher in fragmented forests than in continuous forests. This result aligns with findings from other host and geographic systems and with other parasite groups, such as the gastrointestinal parasites of gray mouse lemurs (e.g., Raharivololona & Ganzhorn, 2009). However, the effects of fragmentation on parasite prevalence have not been consistent across studies. For example, some studies with birds have reported no difference in prevalence between fragmented and continuous forests (e.g., Niu, 2007; Ogrzewalska et al., 2011), whereas another study with skinks found the prevalence to be lower in fragments (e.g., Resasco et al., 2019); further, these effects can vary based on external factors, such as the time of year when animals are sampled (Johnstone et al., 2010). The diverse findings of studies included in our meta‐analysis may reflect similar idiosyncrasies. For example, relative to continuous forests, Bolivar‐Cime et al. (2018) found higher ectoparasite prevalence in fragments, and Hiller et al. (2020) found lower ectoparasite prevalence in fragments surrounded by agriculture (but, interestingly, higher prevalence in fragments surrounded by water) when sampling the Jamaican fruit bat, *Artibeus jamaicensis*. Variation in responses to fragmentation can exist even within a single species (e.g., sex differences; Frank et al., 2016). These often‐conflicting results suggest that the effects of fragmentation on prevalence can be species‐, trait‐, and/or context‐dependent. Thus, although our results suggest that fragmentation increases ectoparasite prevalence in bats overall, there is tremendous value in identifying the specific factors that influence parasite outcomes in fragmented landscapes.

Our inclusion of moderator variables representing bat ecology further supports context dependency of relationships between forest fragmentation and bat ectoparasitism. For all intensity outcomes, the wing aspect ratio was a consistently important predictor. In part, this result can likely be explained by the fact that bats with high wing aspect ratios can physically harbor more ectoparasites because they are larger than those with lower wing aspect ratios (Norberg & Rayner, 1987). Indeed, for phyllostomid bats (which comprised the vast majority of species in our dataset), correlations have been observed between body mass and both the mean and variance of bat fly intensities (Patterson et al., 2008). However, the effects of wing aspect ratio were not always straightforward: Although greater wing aspect ratios are associated with higher ectoparasite intensity variance in continuous forests, the wing aspect ratio has a negative relationship with ectoparasite intensity variance in forest fragments. Mobility differences between species with high and low wing aspect ratios could provide an explanation for this result. For instance, although bats with high wing aspect ratios can more easily disperse to other, less infested fragments than can bats with low wing aspect ratios, they likely have more opportunities to encounter novel, highly infested forest fragments (Luo et al., 2019). However, this mechanism would not explain why there was no difference in *mean* intensity between fragmented and continuous forests. For this, an important consideration is that some ectoparasites (including bat flies) likely do not cause physical damage to their hosts, and the costs of parasitism seem to be limited to primarily the energetic costs of grooming (Dick & Patterson, 2006). It is possible that only hosts with very high intensities would attempt dispersing to avoid or alleviate infestation; in this case, intensity variance could be lower in fragments for bats with greater wing aspect ratios despite similar mean intensities. To our knowledge, however, no study has yet investigated this relationship between bat mobility, the fitness costs of ectoparasites, and intensity variance, and future research is thus needed to better understand this initial pattern.

Consistent with past research, the prevalence was significantly affected by roost duration (e.g., Hiller et al., 2020; Patterson et al., 2007). This result is not surprising, given what is known about bat ectoparasite ecology. Bat flies pupate in roosts and search for bat hosts, whereas mites rely on direct contact between hosts for transmission (Dick & Patterson, 2006; Reckardt & Kerth, 2009). For both parasite groups, higher roost duration would likely facilitate increased transmission. Interestingly, however, mean intensity was not associated with roost duration, which contrasts with past findings where the mean intensity was higher in more permanent structures (Hiller et al., 2020; Patterson et al., 2007). The encounter‐dilution effect (parasitism risk decreasing with increasing group size: Mooring & Hart, 1992; Cote & Poulin, 1995; Rifkin et al., 2012) provides one possible explanation. In our intensity dataset (but not our prevalence dataset), roost duration was highly associated with colony size. For mobile parasites such as bat flies, larger host group sizes are negatively correlated with mean intensity (Patterson & Ruckstuhl, 2013). Intensity variance was similarly unaffected by roost duration across ectoparasites but was affected with mites excluded. Given the different transmission methods of bat flies and mites (i.e., bat flies are mobile, mites are typically not), this result also provides support for the encounter‐dilution effect, as well as context dependence: Although infestation risks could decrease per individual colony member, some of those members could still be more highly parasitized than others.

The *I* ^2^ statistic provides an estimate of true heterogeneity among effect sizes (relative to noise) and is typically high in ecological meta‐analyses (Senior et al., 2016). As expected, we indeed observed high heterogeneity for all three ectoparasite outcomes. Our random effects allow us to decipher the extent to which these factors contribute to observed heterogeneity. For intercept‐only and forest‐type‐only models, heterogeneity was most substantially explained by the random effect of bat phylogeny for all ectoparasite outcomes. Consistent with this, for the forest‐type‐only models, the phylogenetic signal (*H* ^2^) was moderate‐to‐high for all ectoparasite outcomes. However, for the intercept‐only models, *H* ^2^ was relatively low for prevalence and intensity variance (28% and 31%, respectively), suggesting that bat evolutionary history does not explain the vast majority of residual variation for these outcomes. For the univariate forest‐type models, there was a strong phylogenetic signal across all outcomes, suggesting that more closely related bat species have more similar parasite outcomes in response to fragmentation. Important to consider, however, is that these results are likely contingent on the biases that we observed in our literature search on tropical bat parasites (see the *Overall and geographic biases* and *Species biases* sections above).

### 4.3 Providing quantitative measures of fragmentation

One study in our analysis provided a unique description of the habitats that were sampled; all sites experienced some degree of fragmentation, but some could still be considered continuous forest depending on when the land was last cleared (Orta‐Pineda et al., 2020). After excluding this study from our meta‐analysis, we observed similar results for the moderator variables (notably, the wing aspect ratio was still an important predictor for all outcomes), yet the overall effects of forest type on parasite outcomes were weaker. Although this could suggest the true effect of forest type on parasite outcomes was different within this particular study, such sensitivity could as easily be attributed to its larger sample size (i.e., the number of unique records) relative to our remaining studies; as such, removal of this study entirely from our analyses could negatively affect our ability to detect any initial trends.

The challenges associated with sensitivity to study inclusion or exclusion here highlight a future need for researchers to provide quantitative, rather than qualitative, measures of forest fragmentation. Indeed, almost all studies in our analysis described some degree of human disturbance, even in the continuous forests, and fragments also varied in both their size and their level of disturbance (e.g., whether sites were used for agriculture). Quantitative estimates of fragmentation (e.g., patch size or anthropogenic footprint) would improve comparability among studies. Further, any degree of disturbance could result in different researchers assigning different binary categories to a particular site, and quantitative information would alleviate some aspects of this potential bias in future meta‐analyses of fragmentation and parasite outcomes.

Quantitative fragmentation measures could also facilitate the inclusion of a broader range of habitat types in future meta‐analyses, thereby avoiding restricting studies to binary (i.e., “fragmented” or “continuous”) sampling locations. This is likely relevant for many researchers who are logistically limited to particular sites. One such logistical barrier could be that truly pristine forests are often limited to protected areas that frequently require additional permits. From another perspective, some bat species are simply more abundant in disturbed or fragmented areas. For instance, common vampire bats (*Desmodus rotundus*) feed on livestock and are often found in agricultural landscapes (Becker, Czirják, et al., 2018; Bobrowiec et al., 2015); researchers investigating the effects of fragmentation on *D. rotundus* parasites may thus be especially limited to sampling only fragmented areas that will vary in patch size or disturbance. Providing quantitative fragmentation measures could allow such studies to be more easily compiled and compared in quantitative analyses.

To report quantitative habitat data, we recommend complementing publicly available satellite data with field measurements. For example, the Global Forest Cover Change (Hansen et al., 2013) dataset can be used to quantify forest cover loss or gain at a given location, and the Global Human Footprint (Venter et al., 2018) and Global Human Modification of Terrestrial Systems (GMTS; Kennedy et al., 2020) datasets can be used to quantify anthropogenic disturbances. However, because satellite data are typically coarse‐scale, researchers must also provide fine‐scale descriptions of each site. Specifically, authors should report: (1) percent forest cover, (2) patch size, (3) distance to other patches, and (4) successional stage. Although not necessarily quantitative, other information that could provide insight into the magnitude of fragmentation effects should also be reported (e.g., for agriculture: crops/livestock or monoculture/biodiverse; for environmental features: natural/human‐made), including the source of fragmentation (i.e., the disturbance type). For fragments that are anthropogenic in nature, the five input categories from the GMTS dataset could be used to categorize the disturbance type (with some quantitative metrics suggested in parentheses): human settlement (population size), agriculture (patch size), transportation (road size/frequency of use), mining/energy production, and electric infrastructure (Kennedy et al., 2020). Finally, to facilitate comparisons among studies, authors should publish GPS coordinates for their sites. Access to coordinates can allow other researchers to compare sites among studies using satellite data, facilitating more research, and providing deeper insight into the effects of fragmentation (or other disturbances) on bat ectoparasites.

## 5 CONCLUSIONS

Although the association between forest fragmentation and bat ectoparasites has been increasingly studied (e.g., Bolívar‐Cimé et al., 2018), research is still limited. In particular, our gap analysis highlights several striking geographic and taxonomic biases in the current literature, including that studies are almost exclusively limited to the Neotropics and are largely conducted with phyllostomid bats. Thus, in addition to calling for more research overall, an obvious next step is to conduct studies in other regions of the tropics and with a wider range of species. Particularly interesting for comparative studies of habitat fragmentation and bat ectoparasites would be cosmopolitan bat families, such as the Molossidae or Vespertilionidae. Because these families are globally distributed, research on mollosids and vesper bats could provide substantial insight into whether the effects of fragmentation on bat ectoparasites vary geographically. Researching these (and other) widely distributed bat families will be necessary to draw broad conclusions about the effects of habitat comparisons on bat ectoparasites; at present, owing to strong biases towards phyllostomid bats, it is unclear whether current trends are generalizable to all bats, to only Neotropical bats, or to only phyllostomid bats.

Our preliminary meta‐analysis results suggest that at least some aspects of Neotropical bat ectoparasitism are affected by forest fragmentation, and this could help to better understand bat health and manage bat conservation across fragmented and degraded landscapes. Our results also point to the importance of context and trait dependencies in dictating the outcome of bat ectoparasite responses to fragmentation. We thus suggest that identifying the extent to which various traits and/or contexts contribute to variation in these responses would be highly valuable. Our goal was to quantify an overall effect size for the relationship between forest fragmentation and ectoparasite outcomes across ecological and evolutionary contexts and study idiosyncrasies. Identifying additional sources of variation that can affect the relationship between fragmentation and ectoparasite outcomes could thus allow future meta‐analyses to consider even more potential moderators of the relationship between fragmentation and ectoparasite outcomes (e.g., sex, age, matrix type), and identify new and interesting hypotheses about this relationship that current studies are not yet considering. Future research on how habitat fragmentation affects a diverse range of parasites in bats is critical, owing to the overall lack of research in this area (reflected in the small sample size of our analysis), and will be increasingly important as humans continue to degrade and fragment tropical forests, with important consequences for both bat health and conservation.

## AUTHOR CONTRIBUTIONS

**Alexis Heckley:** Conceptualization (equal); data curation (lead); formal analysis (equal); visualization (equal); writing – original draft (lead); writing – review and editing (equal). **Daniel Becker:** Conceptualization (equal); formal analysis (equal); supervision (lead); visualization (equal); writing – review and editing (equal).

## FUNDING INFORMATION

This work was supported by the NSERC CREATE in Biodiversity, Ecosystem Services and Sustainability (BESS). Financial support was provided by the University of Oklahoma Libraries' Open Access Fund.

## CONFLICT OF INTEREST STATEMENT

The authors declare no conflicts of interest.

### OPEN RESEARCH BADGES

This article has earned an Open Data badge for making publicly available the digitally‐shareable data necessary to reproduce the reported results. The data is available at https://doi.org/10.5061/dryad.34tmpg4px.

## Supporting information


Appendix S1.

Appendix S2.

## Data Availability

Data are archived in the Dryad data repository: https://doi.org/10.5061/dryad.34tmpg4px.
