# Supplementary material for: Tropical bat ectoparasitism in continuous versus fragmented forests: A gap analysis and preliminary meta‐analysis
Source: Ecol Evol. 2023 Feb 1;13(2):e9784. doi: 10.1002/ece3.9784 (PMC9891993; doi:10.1002/ece3.9784)
Supplement: Supplementary file 1 — Appendix S1. [file ECE3-13-e9784-s002.docx]

**Tropical bat ectoparasitism in continuous versus fragmented forests: a gap analysis and preliminary meta-analysis**

**Appendix S1 - Data sources:**

***Included in both gap and meta-analysis:***

Bezerra, R.H.S., de Vasconcelos, P.F., Bocchiglieri, A., 2016. Ectoparasites of bats (Mammalia: Chiroptera) in Atlantic forest fragments in north-eastern Brazil. Parasitol Res 115, 3759–3765. <https://doi.org/10.1007/s00436-016-5137-8>

Bolívar-Cimé, B., Cuxim-Koyoc, A., Reyes-Novelo, E., Morales-Malacara, J.B., Laborde, J., Flores-Peredo, R., 2018. Habitat fragmentation and the prevalence of parasites (Diptera, Streblidae) on three Phyllostomid bat species. Biotropica 50, 90–97. <https://doi.org/10.1111/btp.12489>

Frank, H.K., Mendenhall, C.D., Judson, S.D., Daily, G.C., Hadly, E.A., 2016. Anthropogenic impacts on Costa Rican bat parasitism are sex specific. Ecol Evol 6, 4898–4909. <https://doi.org/10.1002/ece3.2245>

Hiller, T., Brändel, S.D., Honner, B., Page, R.A., Tschapka, M., 2020. Parasitization of bats by bat flies (Streblidae) in fragmented habitats. Biotropica 52, 488–501. <https://doi.org/10.1111/btp.12757>

Orta-Pineda, G., Rodríguez-Valencia, V.M., Rico-Chávez, O., Zamora-Bárcenas, D.F., Rodríguez-Moreno, Á., Montiel-Parra, G., Arenas-Montaño, M., Galindo-Maldonado, F.A., Suzán-Azpiri, G., Ojeda-Flores, R., 2020. Composición de comunidades y filoespecificidad de ectoparásitos de murciélagos en paisajes agropecuarios de Veracruz, México. Ecosist. Recur. Agropec. 7. <https://doi.org/10.19136/era.a7n1.2059>

***Included in gap analysis only:***

Hernández-Martínez, J., Morales-Malacara, J.B., Alvarez-Añorve, M.Y., Amador-Hernández, S., Oyama, K., Avila-Cabadilla, L.D., 2019. Drivers potentially influencing host–bat fly interactions in anthropogenic neotropical landscapes at different spatial scales. Parasitology 146, 74–88. <https://doi.org/10.1017/S0031182018000732>

Phelps, K.L., Kingston, T., 2018. Environmental and biological context modulates the physiological stress response of bats to human disturbance. Oecologia 188, 41–52. <https://doi.org/10.1007/s00442-018-4179-2>

Ralisata, M., Andriamboavonjy, F.R., Rakotondravony, D., Ravoahangimalala, O.R., Randrianandrianina, F.H., Racey, P.A., 2010. Monastic Myzopoda : the foraging and roosting ecology of a sexually segregated Malagasy endemic bat. Journal of Zoology 282, 130–139. <https://doi.org/10.1111/j.1469-7998.2010.00724.x>

Ramalho, D.F., Diniz, U.M., Aguiar, L.M.S., 2021. Anthropization Affects the Assembly of Bat-Bat Fly Interaction Networks. Front. Environ. Sci. 9, 752412. https://doi.org/10.3389/fenvs.2021.752412
