# Supplementary material for: Tropical bat ectoparasitism in continuous versus fragmented forests: A gap analysis and preliminary meta‐analysis
Source: Ecol Evol. 2023 Feb 1;13(2):e9784. doi: 10.1002/ece3.9784 (PMC9891993; doi:10.1002/ece3.9784)
Supplement: Supplementary file 2 — Appendix S2. [file ECE3-13-e9784-s001.docx]

**Appendix S2: Tropical bat ectoparasitism in continuous versus fragmented forests: a gap analysis and preliminary meta-analysis**


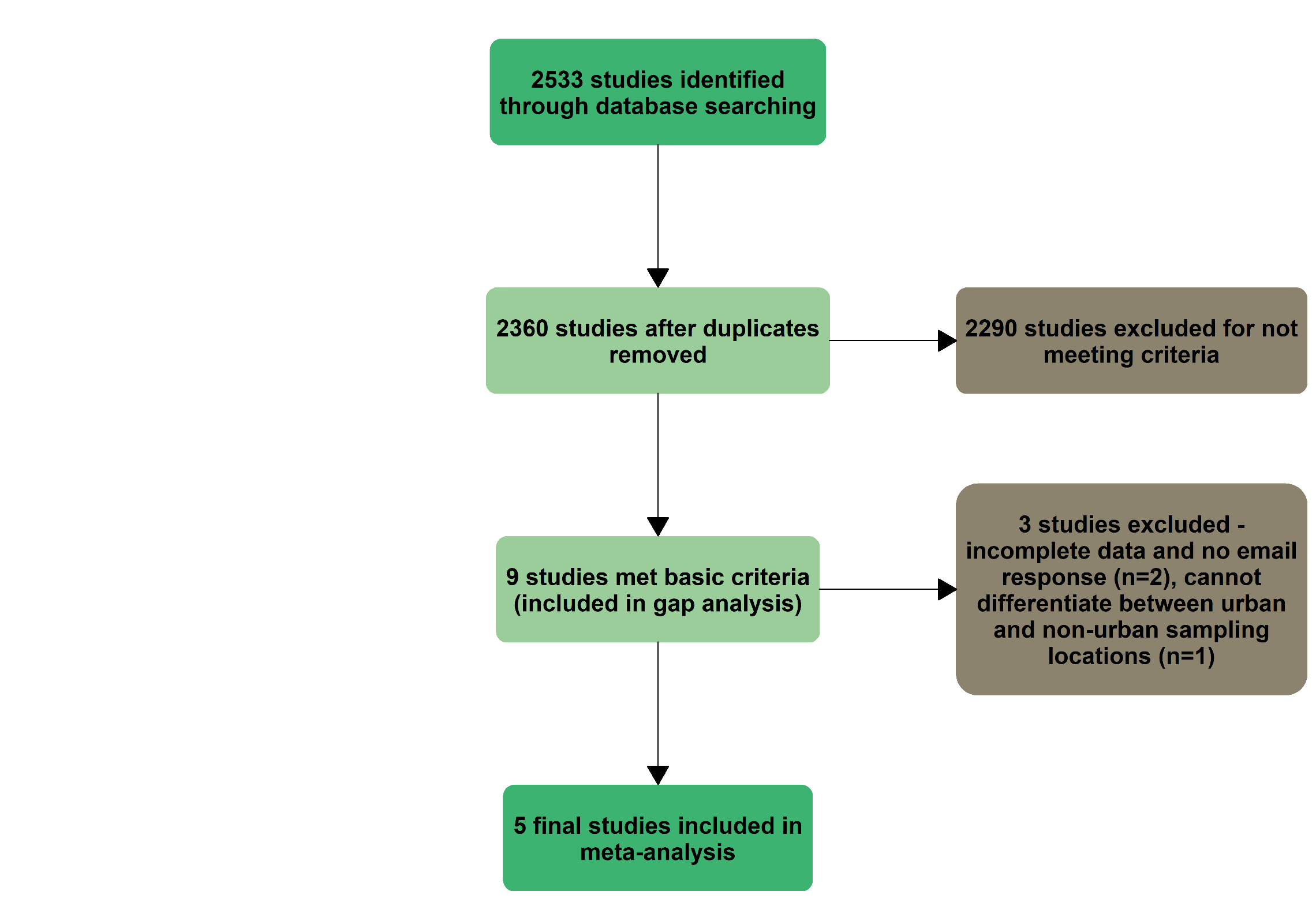


Figure A1 PRISMA diagram for studies included in the meta-analysis.

Table A1 Forest descriptions from the original studies that we used to categorize sites as ‘fragmented’ or ‘continuous’ for use in the meta-analysis.

|  | Study | Fragmented | Continuous |
| --- | --- | --- | --- |
| **Included in gap and meta-analysis:** | Bezzera et al. 2016 | “[…] measures approximately 766 ha (Malta et al. 2011). It consists of patches with different arboreal strata resulting from the human actions of intensive logging and monoculture of sugar cane." | “[…] an area of transition of Atlantic forest, Caatinga in Sergipe, and measures 7966 ha (Costa 2014). Its vegetation consists of forest areas (primary or secondary forest), natural open areas (white sand and wet or dry fields of grasses), and disturbed open areas (exposed soil and crops) (Dantas and Ribeiro 2010). |
|  | Bolivar-Cime et al. 2018 | "[...] dominated by induced pastures for livestock production, where the forest cover (tropical semi-deciduous forest and secondary vegetation) is limited to numerous small patches” | “There are relatively extensive areas with a landscape matrix dominated by continuous forest vegetation (tropical semi-deciduous forest) with some small cleared patches intended for seasonal agriculture.” |
|  | Frank et al. 2016 | “[...] located in coffee plantations with ~5–25% local tree cover on the farms” | "Protecting roughly 280 ha of primary and mature secondary premontane tropical wet forest (Holdridge 1967), the Las Cruces Biological Reserve lies in the Coto Brus Valley surrounded largely by pasture, cropland, houses, and remnant forest fragments (Mendenhall et al. 2014)."  “Bats were sampled in 18 sites of varying tree cover; 12 forested sites included riparian remnant forests, small forest fragments, secondary forest, and forest reserve sites (~25–77% tree cover at a 1000 m radius).”* |
|  | Hiller et al. 2019 | “Forest fragments surrounded by water, and forest fragments in an agricultural matrix” | “Continuous forest” |
|  | Orta-Pineda et al. 2020 | “Induced grassland was considered to be those sites whose landscape was dominated by the presence of forage grasses; silvopastoral to sites where a combination of forage grasses, arboreal legumes and shrubs dominate the landscape” | “[…] acahual [are] sites with landscapes dominated by secondary vegetation derived from a tall evergreen forest that includes the presence of trees, shrubs, herbaceous and grasses.” |
| **Included in gap analysis only** | Hernández-Martínez et al. 2019 | “For this study, we selected 10 sampling plots (8 secondary forest and 2 old growth forest) in and around the Chamela-Cuixmala Biosphere Reserve (CCBR), representing a gradient of vegetation structural complexity and of the degree of preservation of the original vegetation coverage (a forest cover and fragmentation gradient)” | |
|  | Phelps and Kingston, 2018 | “Caves were selected across a gradient of human disturbance, from undisturbed caves in protected areas to moderately disturbed caves exploited by local residents to caves subject to high levels of disturbance from active mining and tourism.” | |
|  | Ralisata et al. 2010 | “Field surveys revealed the following habitat types:  1. Coffee plantation: mainly coffee bushes (*Mascarocoffea sp., Arabusta sp., and Canephora sp.*), with *R. madagascariensis* and other large trees.  2. Degraded lowland humid forest: consisting mainly of *R. madagascariensis*, a few large trees and bamboo.  3. Intact humid forest: little evidence of tree felling, fire and invasion by *R. madagascariensis*.  4. Wooded grassland: includes open sites, with grass, some bamboo and fruit trees (litchis, mangoes).  5. Rice paddy: occurs throughout the valley.  6. Banana field: either as a monoculture or combined with coffee.  7. Habitation: the small town of Kianjavato” | |
|  | Ramalho et al. 2021 | “For each sampling site, we defined a 3-km buffer and quantified the percentage of natural and anthropized area within that radius.” […] “Areas consisting of agriculture, construction, mining, roads, or any man-made structure were considered anthropized, while areas with natural vegetation of any type (e.g., savanna, grasslands, and gallery forests) were considered natural.” | |

*Note that we only included sites with >50% forest cover at 100 and 1000 metres in an attempt to include only the most continuous forests.

Table A2 Complete list of AIC(c) values for all candidate models.

| Outcome variable | Formula | k | delta | wi |
| --- | --- | --- | --- | --- |
| Prevalence | forest type * wing aspect ratio + forest type * roost duration | 6 | 0 | 0.799761 |
|  | forest type * wing aspect ratio + forest type * new max col size + forest type * roost duration | 8 | 3.632 | 0.130101 |
|  | forest type * wing aspect ratio + forest type * primary diet + forest type * roost duration | 12 | 5.642 | 0.047623 |
|  | forest type * wing aspect ratio + forest type * primary diet + forest type * new max col size + forest type * roost duration | 14 | 9.245 | 0.00786 |
|  | forest type * wing aspect ratio | 4 | 9.932 | 0.005575 |
|  | forest type * roost duration | 4 | 10.278 | 0.004689 |
|  | forest type * wing aspect ratio + forest type * new max col size | 6 | 11.614 | 0.002404 |
|  | forest type | 2 | 13.839 | 0.00079 |
|  | forest type * new max col size + forest type * roost duration | 6 | 14.148 | 0.000677 |
|  | forest type * new max col size | 4 | 16.133 | 0.000251 |
|  | forest type * primary diet + forest type * wing aspect ratio | 10 | 17.163 | 0.00015 |
|  | forest type * wing aspect ratio + forest type * primary diet + forest type * new max col size | 12 | 18.886 | 6.34E-05 |
|  | forest type * primary diet + forest type * roost duration | 10 | 20.42 | 2.94E-05 |
|  | forest type * primary diet | 8 | 21.676 | 1.57E-05 |
|  | forest type * primary diet + forest type * new max col size | 10 | 24.116 | 4.64E-06 |
|  | forest type * primary diet + forest type * new max col size + forest type * roost duration | 12 | 24.523 | 3.78E-06 |
| Mean intensity | forest type * wing aspect ratio + forest type * int primary diet | 6 | 0 | 0.344025 |
|  | forest type * wing aspect ratio + forest type * int primary diet + forest type * roost duration | 8 | 0.633 | 0.250689 |
|  | forest type * wing aspect ratio | 4 | 1.58 | 0.156134 |
|  | forest type * wing aspect ratio + forest type * roost duration | 6 | 1.974 | 0.128216 |
|  | forest type * int primary diet | 4 | 2.511 | 0.098024 |
|  | forest type * int primary diet + forest type * roost duration | 6 | 6.151 | 0.015882 |
|  | forest type | 2 | 8.373 | 0.005229 |
|  | forest type * roost duration | 4 | 10.506 | 0.0018 |
| Intensity variance | forest type * wing aspect ratio + forest type * int primary diet + forest type * roost duration | 8 | 0 | 0.327148 |
|  | forest type * wing aspect ratio + forest type * int primary diet | 6 | 0.002 | 0.326821 |
|  | forest type * wing aspect ratio + forest type * roost duration | 6 | 0.491 | 0.255932 |
|  | forest type * wing aspect ratio | 4 | 3.954 | 0.045305 |
|  | forest type * int primary diet | 4 | 4.724 | 0.030828 |
|  | forest type * int primary diet + forest type * roost duration | 6 | 7.178 | 0.009038 |
|  | forest type | 2 | 9.13 | 0.003406 |
|  | forest type * roost duration | 4 | 10.74 | 0.001523 |

Table A3. Estimated heterogeneity (*I^2^*) and phylogenetic signal (*H^2^*) from the forest type–only models. The sample sizes of the data used in each model are indicated in parentheses. Values are presented as proportions.

| *I^2^* term | Prevalence (*n* = 411) | Mean intensity (*n* = 116) | Intensity variance (*n* = 116) |
| --- | --- | --- | --- |
| Study | 0.05 | 0.00 | 0.00 |
| Observation | 0.24 | 0.18 | 0.36 |
| Bat species | 0.00 | 0.00 | 0.00 |
| Bat phylogeny | 0.60 | 0.77 | 0.60 |
| Total *I^2^* | 0.89 | 0.95 | 0.96 |
| Total *H^2^* | 0.67 | 0.81 | 0.62 |

**Analyses with Orta-Pineda et al. (2020) excluded**

Due to a unique definition of forest fragmentation, we ran identical analyses to those in the manuscript, but with the Orta-Pineda et al. (2020) study excluded. The dataset with Orta-Pineda et al. (2020) excluded consisted of 221 prevalence observations from four countries (Brazil, Costa Rica, Mexico, Panama), representing 41 bat species and at least nine ectoparasite species. 60% of the records were from fragments, of which 71% were used by humans, e.g., for agriculture. There were also 53 intensity observations from two countries (Costa Rica, Panama), representing 14 bat species, and at least one ectoparasite species. For the intensity data, 764% were from fragments, of which 65% were used by humans. Refer to the manuscript for details about outcome variable calculation and statistical analyses.

Table A4 AICc results used for model selection with the Orta-Pineda et al. (2020) study excluded.

| Outcome variable | Formula | k | delta | wi |
| --- | --- | --- | --- | --- |
| Prevalence | forest type * wing aspect ratio + forest type * roost duration | 6 | 0 | 0.72342 |
|  | forest type * wing aspect ratio + forest type * new max col size + forest type * roost duration | 8 | 3.2 | 0.146056 |
|  | forest type * wing aspect ratio | 4 | 5.477 | 0.046782 |
|  | forest type * wing aspect ratio + forest type * primary diet + forest type * roost duration | 12 | 6.857 | 0.023465 |
|  | forest type | 2 | 6.973 | 0.022142 |
|  | forest type * roost duration | 4 | 7.971 | 0.013443 |
|  | forest type * wing aspect ratio + forest type * new max col size | 6 | 8.561 | 0.010009 |
|  | forest type * new max col size | 4 | 9.731 | 0.005576 |
|  | forest type * wing aspect ratio + forest type * primary diet + forest type * new max col size + forest type * roost duration | 14 | 10.191 | 0.00443 |
|  | forest type * new max col size + forest type * roost duration | 6 | 10.257 | 0.004287 |
|  | forest type * primary diet + forest type * wing aspect ratio | 10 | 16.449 | 0.000194 |
|  | forest type * primary diet | 8 | 17.582 | 0.00011 |
|  | forest type * wing aspect ratio + forest type * primary diet + forest type * new max col size | 12 | 20.001 | 3.28E-05 |
|  | forest type * primary diet + forest type * roost duration | 10 | 20.394 | 2.70E-05 |
|  | forest type * primary diet + forest type * new max col size | 10 | 21.07 | 1.92E-05 |
|  | forest type * primary diet + forest type * new max col size + forest type * roost duration | 12 | 23.188 | 6.67E-06 |
| Intensity variance | forest type * wing aspect ratio + forest type * int primary diet | 6 | 0 | 0.448683 |
|  | forest type * wing aspect ratio + forest type * int primary diet + forest type * roost duration | 8 | 1.149 | 0.252603 |
|  | forest type * wing aspect ratio + forest type * roost duration | 6 | 3.279 | 0.087079 |
|  | forest type * wing aspect ratio | 4 | 3.368 | 0.083289 |
|  | forest type * int primary diet | 4 | 3.601 | 0.07413 |
|  | forest type | 2 | 5.634 | 0.026825 |
|  | forest type * int primary diet + forest type * roost duration | 6 | 6.966 | 0.013781 |
|  | forest type * roost duration | 4 | 6.991 | 0.01361 |
| Mean intensity | forest type * wing aspect ratio + forest type * int primary diet | 6 | 0 | 0.315782 |
|  | forest type * int primary diet | 4 | 0.015 | 0.313423 |
|  | forest type * wing aspect ratio + forest type * int primary diet + forest type * roost duration | 8 | 1.102 | 0.182008 |
|  | forest type * wing aspect ratio + forest type * roost duration | 6 | 3.149 | 0.065402 |
|  | forest type * int primary diet + forest type * roost duration | 6 | 3.281 | 0.061225 |
|  | forest type * wing aspect ratio | 4 | 4 | 0.042736 |
|  | forest type | 2 | 5.976 | 0.015912 |
|  | forest type * roost duration | 4 | 8.998 | 0.003512 |

Table A5 Full results for the final rma.mv() models for all parasite outcomes with the Orta-Pineda et al. (2020) study excluded. All models include an observation-level random effect nested within a study-level random effect, as well as random effects for species and bat phylogeny.

| Outcome variable |  | estimate | se | z | p | lower 95% CI | upper 95% CI |
| --- | --- | --- | --- | --- | --- | --- | --- |
| Prevalence | Intercept | -9.49 | 3.75 | -2.53 | 0.01 | -16.85 | -2.13 |
|  | Fragment | -1.99 | 4.13 | -0.48 | 0.63 | -10.07 | 6.10 |
|  | Wing aspect ratio | 1.24 | 0.59 | 2.12 | 0.03 | 0.09 | 2.39 |
|  | Roost duration | 0.45 | 0.15 | 3.03 | <0.01 | 0.16 | 0.74 |
|  | Fragment : Wing aspect ratio | 0.39 | 0.66 | 0.59 | 0.56 | -0.91 | 1.68 |
|  | Fragment : Roost duration | -0.06 | 0.14 | -0.44 | 0.66 | -0.34 | 0.22 |
| Intensity variance | Intercept | -9.86 | 3.78 | -2.61 | 0.01 | -17.26 | -2.46 |
|  | Fragment | 13.59 | 4.87 | 2.79 | 0.01 | 4.05 | 23.13 |
|  | Wing aspect ratio | 1.60 | 0.63 | 2.55 | 0.01 | 0.37 | 2.83 |
|  | Non-frugivore | -0.15 | 0.61 | -0.25 | 0.80 | -1.35 | 1.05 |
|  | Fragment : Wing aspect ratio | -2.20 | 0.80 | -2.74 | 0.01 | -3.76 | -0.63 |
|  | Fragment : Non-frugivore | 1.66 | 0.81 | 2.05 | 0.04 | 0.07 | 3.24 |
| Mean intensity | Intercept | -4.52 | 2.16 | -2.10 | 0.04 | -8.75 | -0.29 |
|  | Fragment | 5.52 | 2.86 | 1.93 | 0.05 | -0.09 | 11.14 |
|  | Wing aspect ratio | 0.81 | 0.35 | 2.28 | 0.02 | 0.11 | 1.50 |
|  | Non-frugivore | 0.34 | 0.34 | 1.00 | 0.32 | -0.33 | 1.00 |
|  | Fragment : Wing aspect ratio | -0.91 | 0.47 | -1.92 | 0.05 | -1.83 | 0.02 |
|  | Fragment : Non-frugivore | 0.88 | 0.50 | 1.78 | 0.07 | -0.09 | 1.86 |

Table A6. Estimated heterogeneity (*I^2^*) in parasite outcomes and phylogenetic signal (*H^2^*) from each intercept-only model with Orta-Pineda et al. (2020) excluded. Values are presented as proportions.

| *I^2^ term* | Prevalence | Mean intensity | Intensity variance |
| --- | --- | --- | --- |
| Study | 0.03 | 0.00 | 0.00 |
| Observation | 0.22 | 0.08 | 0.28 |
| Bat species | 0.00 | 0.04 | 0.02 |
| Bat phylogeny | 0.69 | 0.86 | 0.68 |
| *Total I^2^* | 0.93 | 0.97 | 0.98 |
| *H^2^* | 0.34 | 0.62 | 0.44 |
|  |  |  |  |

Table A7. Summary of each forest type–only model with the Orta-Pineda et al (2020) study excluded. Random effects for each model include observation nested within study, bat species, and bat phylogeny.

|  |  | Estimate | se | z | p | lower 95% CI | upper 95% CI |
| --- | --- | --- | --- | --- | --- | --- | --- |
| Prevalence | Intercept | 0.127 | 0.816 | 0.156 | 0.876 | -1.471 | 1.726 |
|  | Fragment | 0.170 | 0.177 | 0.961 | 0.337 | -0.177 | 0.517 |
| Mean intensity | Intercept | 0.837 | 0.463 | 1.807 | 0.071 | -0.071 | 1.745 |
|  | Fragment | 0.042 | 0.104 | 0.399 | 0.690 | -0.163 | 0.246 |
| Intensity variance | Intercept | 0.151 | 0.513 | 0.293 | 0.769 | -0.855 | 1.156 |
|  | Fragment | 0.260 | 0.206 | 1.261 | 0.207 | -0.144 | 0.665 |

Table A8. Estimated heterogeneity (*I^2^*) in parasite outcomes and phylogenetic signal (*H^2^*) from each forest-type model. (with Orta-Pineda et al., 2020 excluded). Values are presented as proportions.

| *I^2^* term | Prevalence | Mean intensity | Intensity variance |
| --- | --- | --- | --- |
| Study | 0.01 | 0.00 | 0.00 |
| Observation | 0.21 | 0.09 | 0.32 |
| Bat species | 0.00 | 0.03 | 0.00 |
| Bat phylogeny | 0.69 | 0.85 | 0.65 |
| Total *I^2^* | 0.92 | 0.97 | 0.96 |
| *H^2^* | 0.75 | 0.88 | 0.67 |

**Analyses with mites excluded**

Because mites and bat flies could differ in ways that might affect the relationship between fragmentation and ectoparasite outcomes, we also ran identical analyses to those in the manuscript but with mites excluded. The dataset without mites consisted of 352 prevalence observations from four countries (Brazil, Costa Rica, Mexico, Panama), representing 45 bat species and at least 30 ectoparasite species. 54% of the records were from fragments, of which 80% were used by humans, e.g., for agriculture. There were also 97 intensity observations from three countries (Costa Rica, Panama, Mexico), representing 17 bat species, and at least 15 ectoparasite species. For the intensity data, 57% were from fragments, of which 78% were used by humans. Refer to the manuscript for details about outcome variable calculation and statistical analyses.

Table A9 AICc results used for model selection with mites excluded.

| Outcome variable | Formula | k | delta | wi |
| --- | --- | --- | --- | --- |
| Prevalence | forest type * wing aspect ratio + forest type * roost duration | 6 | 0 | 0.810593 |
|  | forest type * wing aspect ratio + forest type * new max col size + forest type * roost duration | 8 | 3.918 | 0.114293 |
|  | forest type * wing aspect ratio + forest type * primary diet + forest type * roost duration | 12 | 5.327 | 0.056501 |
|  | forest type * wing aspect ratio + forest type * primary diet + forest type * new max col size + forest type * roost duration | 14 | 9.239 | 0.007991 |
|  | forest type * wing aspect ratio | 4 | 10.379 | 0.004519 |
|  | forest type * roost duration | 4 | 10.819 | 0.003626 |
|  | forest type * wing aspect ratio + forest type * new max col size | 6 | 13.28 | 0.001059 |
|  | forest type * new max col size + forest type * roost duration | 6 | 14.433 | 0.000595 |
|  | forest type | 2 | 14.581 | 0.000553 |
|  | forest type * new max col size | 4 | 17.474 | 0.00013 |
|  | forest type * primary diet + forest type * wing aspect ratio | 10 | 18.213 | 8.99E-05 |
|  | forest type * wing aspect ratio + forest type * primary diet + forest type * new max col size | 12 | 21.272 | 1.95E-05 |
|  | forest type * primary diet + forest type * roost duration | 10 | 21.48 | 1.76E-05 |
|  | forest type * primary diet | 8 | 23.127 | 7.71E-06 |
|  | forest type * primary diet + forest type * new max col size + forest type * roost duration | 12 | 25.244 | 2.67E-06 |
|  | forest type * primary diet + forest type * new max col size | 10 | 26.386 | 1.51E-06 |
| Mean intensity | forest type * wing aspect ratio + forest type * int primary diet | 6 | 0 | 0.345168 |
|  | forest type * wing aspect ratio + forest type * int primary diet + forest type * roost duration | 8 | 0.467 | 0.27329 |
|  | forest type * wing aspect ratio | 4 | 1.571 | 0.157359 |
|  | forest type * wing aspect ratio + forest type * roost duration | 6 | 1.598 | 0.155249 |
|  | forest type * int primary diet | 4 | 3.729 | 0.053492 |
|  | forest type * int primary diet + forest type * roost duration | 6 | 7.463 | 0.008269 |
|  | forest type | 2 | 8.214 | 0.00568 |
|  | forest type * roost duration | 4 | 10.888 | 0.001492 |
| Intensity variance | forest type * wing aspect ratio + forest type * int primary diet + forest type * roost duration | 8 | 0 | 0.614597 |
|  | forest type * wing aspect ratio + forest type * roost duration | 6 | 1.89 | 0.238881 |
|  | forest type * wing aspect ratio + forest type * int primary diet | 6 | 3.652 | 0.098985 |
|  | forest type * wing aspect ratio | 4 | 5.43 | 0.040689 |
|  | forest type * int primary diet | 4 | 10.274 | 0.003611 |
|  | forest type * int primary diet + forest type * roost duration | 6 | 11.705 | 0.001766 |
|  | forest type | 2 | 12.699 | 0.001074 |
|  | forest type * roost duration | 4 | 14.693 | 0.000396 |

Table A10 Full results for the final rma.mv() models for all parasite outcomes with mites excluded. All models include an observation-level random effect nested within a study-level random effect, as well as random effects for species and bat phylogeny.

| Outcome variable |  | estimate | se | z | p | lower 95% CI | upper 95% CI |
| --- | --- | --- | --- | --- | --- | --- | --- |
| Prevalence | Intercept | -7.25 | 3.06 | -2.37 | 0.02 | -13.24 | -1.26 |
|  | Fragment | -4.93 | 3.55 | -1.39 | 0.16 | -11.90 | 2.03 |
|  | Wing aspect ratio | 0.88 | 0.48 | 1.84 | 0.07 | -0.06 | 1.81 |
|  | Roost duration | 0.40 | 0.11 | 3.73 | <0.01 | 0.19 | 0.61 |
|  | Fragment:Wing aspect ratio | 0.84 | 0.57 | 1.48 | 0.14 | -0.27 | 1.96 |
|  | Fragment:Roost duration | 0.00 | 0.11 | 0.01 | 0.99 | -0.21 | 0.21 |
| Mean intensity | Intercept | -5.16 | 1.92 | -2.69 | 0.01 | -8.91 | -1.40 |
|  | Fragment | 3.84 | 2.40 | 1.60 | 0.11 | -0.87 | 8.55 |
|  | Wing aspect ratio | 0.90 | 0.31 | 2.88 | <0.01 | 0.29 | 1.51 |
|  | Non-frugivore | 0.33 | 0.26 | 1.29 | 0.20 | -0.17 | 0.83 |
|  | Fragment:Wing aspect ratio | -0.63 | 0.39 | -1.60 | 0.11 | -1.40 | 0.14 |
|  | Fragment:Non-frugivore | 0.48 | 0.32 | 1.52 | 0.13 | -0.14 | 1.10 |
| Intensity variance | Intercept | -14.78 | 3.59 | -4.11 | <0.01 | -21.82 | -7.73 |
|  | Fragment | 16.46 | 4.70 | 3.51 | <0.01 | 7.26 | 25.67 |
|  | Wing aspect ratio | 2.32 | 0.57 | 4.08 | <0.01 | 1.21 | 3.43 |
|  | Roost duration | 0.26 | 0.11 | 2.42 | 0.02 | 0.05 | 0.47 |
|  | Non-frugivore | -0.67 | 0.45 | -1.47 | 0.14 | -1.56 | 0.22 |
|  | Fragment:Wing aspect ratio | -2.63 | 0.74 | -3.55 | <0.01 | -4.09 | -1.18 |
|  | Fragment:Roost duration | -0.10 | 0.15 | -0.67 | 0.50 | -0.39 | 0.19 |
|  | Fragment:Non-frugivore | 1.73 | 0.67 | 2.57 | 0.01 | 0.41 | 3.04 |

Table A11. Estimated heterogeneity (*I^2^*) in parasite outcomes and phylogenetic signal (*H^2^*) from each intercept-only model with mites excluded. Values are presented as proportions.

| I^2^ term | Prevalence | Mean Intensity | Intensity Variance |
| --- | --- | --- | --- |
| Study | 0.06 | 0.02 | 0.00 |
| Observation | 0.23 | 0.13 | 0.34 |
| Bat species | 0.00 | 0.00 | 0.00 |
| Bat phylogeny | 0.62 | 0.82 | 0.63 |
| Total I^2^ | 0.91 | 0.96 | 0.97 |
| H^2^ | 0.25 | 0.64 | 0.00 |

Table A12. Summary of each forest type–only model with mites excluded. Random effects for each model include observation nested within study, bat species, and bat phylogeny.

|  |  | estimate | se | z | p | lower 95% CI | upper 95% CI |
| --- | --- | --- | --- | --- | --- | --- | --- |
| Prevalence | Intercept | 0.18 | 0.78 | 0.23 | 0.82 | -1.35 | 1.71 |
|  | Fragment | 0.27 | 0.14 | 1.96 | 0.05 | 0.00 | 0.54 |
| Mean Intensity | Intercept | 0.82 | 0.41 | 2.02 | 0.04 | 0.02 | 1.63 |
|  | Fragment | 0.04 | 0.08 | 0.45 | 0.65 | -0.12 | 0.19 |
| Intensity Variance | Intercept | 0.30 | 0.52 | 0.57 | 0.57 | -0.73 | 1.32 |
|  | Fragment | 0.17 | 0.15 | 1.09 | 0.28 | -0.13 | 0.46 |

Table A13. Estimated heterogeneity (*I^2^*) in parasite outcomes and phylogenetic signal (*H^2^*) from each forest-type model (with mites excluded). Values are presented as proportions.

| *I^2^* term | Prevalence | Mean intensity | Intensity variance |
| --- | --- | --- | --- |
| Study | 0.05 | 0.01 | 0.00 |
| Observation | 0.22 | 0.13 | 0.33 |
| Bat species | 0.00 | 0.00 | 0.00 |
| Bat phylogeny | 0.63 | 0.831 | 0.63 |
| Total *I^2^* | 0.91 | 0.96 | 0.96 |
| *H^2^* | 0.70 | 0.85 | 0.66 |
